# Editorial: Dermatologic manifestations of primary immune deficiency disorders in children

**DOI:** 10.3389/fped.2023.1182474

**Published:** 2023-03-31

**Authors:** Oded Shamriz, Shanmuganathan Chandrakasan

**Affiliations:** ^1^ Allergy and Clinical Immunology Unit, Department of Medicine, Hadassah Medical Organization, Faculty of Medicine, Hebrew University of Jerusalem, Jerusalem, Israel; ^2^ The Lautenberg Center for Immunology and Cancer Research, Institute of Medical Research Israel-Canada, Faculty of Medicine, Hebrew University of Jerusalem, Jerusalem, Israel; ^3^ Department of Pediatrics, Aflac Cancer and Blood Disorders Center, Children's Healthcare of Atlanta, Emory University School of Medicine, Atlanta, GA, United States

**Keywords:** immune deficiency, skin, dermatologic, manifestations, immune dysregulaiton

**Editorial on the Research Topic** Dermatologic manifestations of primary immune deficiency disorders in children

The introduction of genetic-based diagnosis incorporated with advanced molecular workup has revolutionized the field of primary immune deficiency disorders (PIDD). Number of PIDD is constantly increasing and the 2022 International Union of Immunological Societies (IUIS) update classifies 485 genetic disorders as PIDD (1). Corresponding with advances in PIDD diagnoses, the clinical spectrum of each PIDD is also expanding.

The skin is a major site affected by PIDD. Chronic mucocutaneous candidiasis can be seen in a variety of PIDD including *Signal Transducer and Activator of Transcription* (*STAT)1* gain-of-function (GOF) and dominant negative *STAT3* variants, both characterized by impaired T helper (Th)17 immunity (2). Recurrent staphylococcal skin infections and abscesses are a well-known feature of hyper IgE syndrome (HIES) and chronic granulomatous disease (CGD) (3). In addition, PIDD can lead to unexpected skin infections, as seen in *STAT1* GOF, which was reported to induce rosacea and chronic demodicosis of the skin (4). However, non-infectious dermatological presentation can also be seen in PIDD categorized as primary immune regulatory disorders (PIRD). For example, the classic severe combined immune deficiency (SCID)-related Omenn's syndrome has a severe eczema, which is induced by clones of autoreactive T cells. Autoimmune manifestations of the skin can also be found in other PIRD, such as Wiskott-Aldrich syndrome (WAS), LPS responsive beige-like anchor protein (LRBA) deficiency and cytotoxic T-lymphocyte-associated protein (CTLA)-4 haploinsufficiency (5). In addition, Atopic dermatitis (AD), due to skewed immunity towards Th2 response, can be seen in dominant negative *STAT*3 variants. AD characterizes other PIDD with skewed Th2 immunity, such as DOCK8 deficiency and CARD11-associated atopy with dominant interference of NF-kB signaling (CADINS) syndrome.

Indeed, many of the children with PIDD are first being evaluated by dermatologists, rather than clinical immunologists. Thus, special attention should be given to skin involvement in PIDD.

This issue of *Frontiers in Pediatrics* focuses on dermatologic manifestations of PIDD in children. Different articles presented in this research topic offer a broad view of the subject.

Sarika et al. describes an enigmatic 8-year-old child presenting with acrofacial skin necrosis. Suspecting PIDD involving interferon immunity, immune and genetic workups were initiated, although no definite diagnosis was achieved. Shen et al. reviews the expanding clinical spectrum of inborn errors of NF-κB, with a special focus on skin manifestations and details regarding underlying mechanisms. The authors describe the different phenotypes, including ectodermal dysplasia, impaired quantities of keratinocytes and skin infiltration of inflammatory cells, *via* enhanced tumor necrosis factor (TNF) response. They offer differential diagnosis and suggest a genotype-phenotype correlation as diagnostic clues of inborn errors of NF-κB. Ollech et al. describe eight children with PIDD and unique dermatological presentations. This interesting cohort includes patients with common variable immunodeficiency, (CVID), SCID, DOCK8 deficiency, ataxia telangiectasia, CARD11 deficiency, MALT1 deficiency and CGD. Among unusual infectious dermatological manifestations in these children, they detail ulcerative-hemorrhagic varicella-zoster virus, atypical fungal and bacterial infections, Norwegian scabies, giant perianal verrucae and diffuse molluscum contagiosum. Huang et al. report an interesting association between interleukin (IL)-6 polymorphism and AD. This study comprises of investigation of 132 patients with AD and 100 healthy controls and found that A/G genotype of IL-6 increases the risk for development of AD, thus perhaps aiding in unveiling new methods of genetic workup for children with AD. Finally, Giancotta et al. offers an extensive review on tailored treatment for PIDD associated with atopy. This review nicely demonstrates the notion of personalized medicine, which has a significant place in the treatment of children with PIDD presenting with skin manifestations.

In conclusion, dermatological manifestations of PIDD in children are common and may constitute the initial presenting feature. Collaboration between general pediatricians, dermatologists and clinical immunologists is needed to initiate prompt immunogenetic evaluation and offer better medical care for these children.
